# Factor associated with the occurrence of epilepsy in autism: a systematic review

**DOI:** 10.1007/s10803-022-05672-2

**Published:** 2022-07-29

**Authors:** Eleni Zarakoviti, Roz Shafran, David Skuse, Amy McTague, Neha Batura, Tom Palmer, Emma Dalrymple, Sophie D. Bennett, Colin Reilly

**Affiliations:** 1 UCL Great Ormond Street Institute of Child Health (ICH), 30 Guilford Street, WC1N 1EH London, UK; 2 Great Ormond Street Hospital for Children NHS Trust, Great Ormond Street, WC1N 3JH London, UK; 3 UCL Institute for Global Health (IGH), 30 Guilford Street, WC1N 1EH London, UK; 4 Research Department, Young Epilepsy, RH7 6PW Lingfield, Surrey UK

**Keywords:** Autism, Epilepsy, Children, Adults, Intellectual disability

## Abstract

This systematic review aimed to identify factors significantly associated with the occurrence of epilepsy in autistic individuals and to consider the impact of study quality on findings. Electronic databases were systematically searched on October 2nd, 2020 and records retrieved were limited to those published from 2000 onwards. Study quality was categorised as ‘good’, ‘moderate’ or ‘weak’. Fifty-three studies were included and in studies where the prevalence of epilepsy was reported (n = 257,892), 18,254 (7%) had co-occurring epilepsy. Intellectual disability/cognitive impairment was the most commonly reported risk factor associated with occurrence of epilepsy in autistic individuals. The evidence supporting other, potentially relevant factors was weak and inconsistent and requires further evaluation. Only 9/53 studies were considered ‘good’ quality.

Medical comorbidities are more prevalent in autistic individuals than those without autism (Kohane et al., 2012), especially neurological conditions (Pan et al., 2020) of which epilepsy is the most common (Pan et al., 2020); affecting between 12.1% and 17.2% (Lukmanji et al., 2019; Pan et al., 2020) compared with 0.5-1% in the general population. Prevalence estimates vary because studies have used different definitions of epilepsy/autism and assessment methods, but if epilepsy is present it is associated with increased mortality (Hirvikoski et al., 2016) and relatively high health care costs (Peacock et al., 2012). Studies of individuals with epilepsy have also noted an increased prevalence of autism (Strasser et al., 2018).

Epilepsy is a disease of the brain typically defined by the presence of two unprovoked (or reflex) seizures occurring > 24 h apart or the presence of an epilepsy syndrome (Fisher et al., 2014). The point prevalence of active epilepsy is 6.38 per 1,000 persons while the lifetime prevalence was 7.60 per 1,000 persons (Fiest et al., 2017). The prevalence and incidence rate of epilepsy are higher in low to middle income countries (Fiest et al., 2017).

Understanding factors associated with the co-occurrence of epilepsy in autistic individuals may improve identification of epilepsy in autistic individuals and may also aid in defining biological subtypes within autism and in the development of specific targeted interventions (Tye et al., 2019). A previous systematic review and meta-analysis undertaken in 2008 noted that epilepsy in autistic individuals was associated with the presence of intellectual disability and female gender (Amiet et al., 2008). Since this review was published new diagnostic criteria for autism have been published (American Psychiatric Association, 2013). Additionally, the prevalence and incidence of registered diagnoses of autism have increased significantly (Lundström et al., 2015; Russell et al., 2021) in the last 2 decades. These increases likely reflect changes in reporting and how diagnoses are applied and could have an impact on the reported association between epilepsy and autism with respect to associated factors. Thus, the aim of the present systematic review is to identify factors associated with the co-occurrence of epilepsy in autism since 2000. The impact of the quality of the studies assessed on the findings of the present review is also assessed.

## Methods

### Search Strategy

The current methodology used is a systematic literature review performed according to the Preferred Reporting Items for Systematic Reviews (PRISMA) guidelines (Liberati et al., 2009; Moher et al., 2009). Electronic databases PsycINFO, Medline, Web of Science, CINAHL and Cochrane were systematically searched on October 2, 2020 using the following search terms: (autis* or ASD or PDD or asperger* or pervasive developmental disorder*) combined with the ‘autism spectrum disorder’ subject heading and (epilep* or seizure*) combined with the ‘epilepsy’ subject heading. The records retrieved were limited to those available in the English language and published from 2000 onwards. In addition to the electronic search, we conducted a hand search of articles included from the electronic search to identify articles that may not have been indexed in the electronic databases.

### Screening of abstracts and full text articles

Records were initially screened by title and abstract by two independent reviewers (CR and EZ) in Covidence (https://app.covidence.org) and then by full text leading to the final set of eligible papers. Predetermined exclusion and inclusion used in both search stages are listed in Supplement 1. Studies that included predominantly participants with neurogenetic syndromes (e.g., Tuberous Sclerosis Complex, Dravet Syndrome, Rett Syndrome) known to have a high association with intellectual disability and the findings of these studies are presented separately (see Supplement 9).

### Data extraction

A data extraction form (Supplement 2) was developed to obtain the sample characteristics and main outcomes linked to the co-occurrence of epilepsy in autism in the eligible papers. Details of all studies included in the present review can be found in Table 1, and 2 referring to case-control studies and observational/cohort/cross-sectional studies respectively. For the purposes of this review, case-control studies were defined as studies that include at least two distinct groups of participants, one with autism and one without. Observational/cross-sectional/cohort studies are defined as studies featuring autistic individuals only differing with regards to particular characteristics (such as age or intellectual capacities) and compared in terms of the presence or lack of co-occurring epilepsy.

**Table 1:** Study Characteristics for observational, cohort & cross-sectional studies

Authors	YR	Country	ASD SS	M	F	AGE (m,r)	Sample Source	ASD + E SS	Epilepsy Prevalence	ASD Definition/ Instruments	Epilepsy Definition (criteria)	Study Quality
Fombonne et al.	2020	UK	2917	2301	616	23.2, (> 18)	Clinical Sample	452	15.5%	DSM IV	Medical Record Review	Moderate
Bishop et al.	2020	USA	7513	1004	471	• ASD alone : 26.7,(< 30 - >60) • ASD + ID: 33.45, (< 30 - >60)	Population Based Sample	1475	19.6%	ICD 9 & 10	ICD 9, ICD 10	Moderate
Gilmore et al.	2020	USA	4685	3175	1510	NR, (> 65)	Clinical Sample	1239	26.4%	ICD 10	ICD 10	Moderate
Waddington et al.	2020	Australia	203	163	40	8.47, (2–18)	Clinical Sample	37	18.2%	ADOS, AQ, SRS	Autism Family Questionnaire	Moderate
Thompson et al.	2019	Sweden	303	254	49	• Sample 1: T1: 3, (19–60 months) T2: 5.2, (19–60 months) • Sample2: T1: 3.8, (19–60 months), T2: 5.5, (19–60 months)	Community Based Sample	44	14.5%	DSM IV, ABC, ADOS-G	Parental Reports	Moderate
Lamb et al.	2019	South Africa	86	64	22	NR, (3–13)	Clinical Sample	20	13.3%	DSM 5	EEG data & Medical History	Moderate
Hwang et al.	2019	Australia	35,929	28,555	170	NR, (5–65)	Clinical Sample	1857	5.2%	ICD 10	ICD 10	Moderate
Miot et al.	2019	France	63	46	17	42.9, (21–68)	Clinical Sample	18	28.6%	DSM 5, CARS	Medical Records Review, Biological Examination & Medical Examinations carried out by the authors	Moderate
Zhang et al.	2018	USA	132,872	107,829	24,985	NR, (3–17)	Clinical Sample	6274	4.7%	ICD-9	ICD 9	Moderate
Kommu et al.	2017	India	201	160	41	5.29, (NR)	Clinical Sample	31	15.4%	ICD 10	ICD 10	Moderate
Gadow et al.	2017	USA	213	157	27	• ASD + regression: 10.8 (NR) • ASD no regression: 10.7 (NR)	Clinical Sample	11	5.1%	DSM IV, CASI-4R Observations, Developmental history, ADOS, Clinical interviews of the child and caregiver & parent questionnaire	The Parent Questionnaire	Moderate
Wise et al.	2017	USA	74	61	13	NR	Clinical Sample	17	23%	DSM 5, Medical records	Medical Record Review	Weak
Wu et al.	2016	USA	7773	NR	NR	NR	Clinical Sample	434	5.6%	DSM IV-R	Statement from a medical professional in the child records	Moderate
Ayta et al.	2016	Turkey	137	115	22	7.6, (2.7–14)	Clinical Sample	8	5.8%	DSM IV, CARS,ABC	Medical History, EEG	Moderate
Christensen et al.	2016	Denmark	186,860	NR	NR	NR	Population Based Sample	NR	NA	ICD 10	ICD 10	Good
Fortuna et al.	2016	USA	255	192	63	33.6, (18–71)	Clinical Sample	30	11.8%	ICD 9	NHIS 2013	Moderate
Shubrata et al.	2014	India	50	39	11	Total mean = 8.23, (3–19)	Clinical Sample	25	50%	DSM IV, PDD Assessment Scale, CARS	ILAE 1981 and 1989	Weak
Saltik et al.	2014	Turkey	121	92	29	9.30, (3–18)	Clinical Sample	40	33.1%	DSM IV-R	• Definition: At least two seizures (febrile or afebrile) after the newborn period (other than those due to acute symptomatic causes) • EEG/ MRI scans.	
Mulligan et al.	2014	USA	101	78	23	7.06, (1.68–18.32)	Clinical Sample	60	59.4%	DSM IV	Medical Record Review	Weak
Schuch et al.	2014	Brazil	209	170	39	9.86, (NR)	Clinical Sample	58	27.8%	DSM IV, ASQ, CARS	Parent/caregiver reports	Moderate
Wong et al.	2014	China	1261	1109	152	• ASD + dysmorphic features: 7.19, (0-32.78) • ASD alone: 6.55, (0-29.90)	Clinical Sample	73	5.8%	DSM IV	Medical Record Review	Moderate
Doshi-Velez et al.	2014	USA	4934	3849	1085	NR	Electronic Health Records	1144	23.2%	ICD 9	Medical Record Review	Moderate
Eriksson et al.	2013	Sweden	208	176	32	NR	Clinical Sample	18	8.7%	DSM IV	Clinical Diagnosis of Epilepsy	Good
Saltik et al.	2012	Turkey	121	92	29	9.30, (3–18)	Clinical Sample	33	27.8%	DSM IV	Parent Reports, Medical records, EEG results	Moderate
Ververi et al.	2012	Greece	222	165	41	NR	Clinical Sample	17	7.7%	DSM IV	Medical records, EEG	Moderate
Bolton et al.	2011	UK	150	104	46	32.61 (26–56)	Clinical Sample	33	22%	ICD 10, ADI-R, ADOS	Epilepsy interview (to the parents) & medical notes	Good
Parmeggiani et al.	2010	Italy	345	277	68	10.5, (2–37 years)	Clinical sample	86	24.9%	DSM-IV-TR CARS	EEG and Medical records	Moderate
Smith et al. A	2010	USA	50	36	14	48.58, (29–72)	Clinical Sample	25	50%	DSM IV-TR & ICD 10	ILAE 1989	Moderate
Smith et al. B	2010	USA	50	36	14	48.58, (29–72)	Clinical Sample	25	50%	DSM IV-TR & ICD 10	ILAE 1989	Moderate
Smith et al. C	2010	USA	50	36	14	48.58, (29–72)	Clinical Sample	25	50%	DSM IV-TR & ICD 10	ILAE 1989	Moderate
Ming et al.	2008	USA	160	131	29	6, (2–18)	Clinical Sample	22	13.8%	DSM IV, ADI-R, ADOS, CARS	Diagnosed via neurological assessment Epilepsy defined as: 2 or more unprovoked seizures	Moderate
Hara	2007	Japan	130	106	24	21*,(18 − 15)	Clinical Sample	33	25%	DSM-IV	ILAE 1993	Moderate
Oslejskova et al.	2007	Czech Repulic	205	145	60	10, (5–15)	Clinical Sample	103	50.2%	ICD 10, CARS & CAST	EEG, Neurological Assessment	Good
Billstedt et al.	2007	Sweden	105	84	36	25.5, (17–40)	Community Based Sample	NR	NA	DSM IV, ICD 10, DISCO, DSM 3-R	Medical Assessment	Good
Danielsson et al.	2005	UK	108	77	31	25.5 (17–40)	Clinical Sample	43	39.8%	DSM IV, DISCO	ILAE 1981	Good
Pavone et al.	2004	Italy	72	57	15	9.04 (4–21)	Clinical Sample	19	26.4%	DSM 3, DSM IV, CARS	• Definition: Two or more recurrent, unprovoked seizures • Tests: EEG, MRI, CT	Good
Fombonne et al.	2004	UK	294	238	56	NR	Clinical Sample	34	11.6%	DSM IV	GPRD & case validation form	Weak
Hashimoto et al.	2001	Japan	2631	2301	330	6, (2.3–19.5)	Clinical Sample	452	17.2%	DSM IV	EEG data	Moderate
Miles et al.	2000	USA	86	69	17	9.5, (1-41.2)	Clinical Sample	30	21.9%	DSM IV, CARS	Medical Record Review	Moderate

• YR: Year, ASD SS: ASD Sample size, M: Males, F: Females, m: Mean, r: Range, ASD SS:ASD Sample size, ASD + E SS: ASD and Epilepsy Sample size, NR: Not reported, NA: Not applicable, *median age not mean age

ICD 8: International Classification of Disease, 8th Edition (World Health Organization, 1971)

ICD 9: International Classification of Disease, 9th Edition (World Health Organization, 1978)

ICD 10: International Classification of Disease, 10th Edition (World Health Organization, 1992)

DSM 3-R: Diagnostic and Statistical Manual of Mental Disorders, 3th Edition Revised (American Psychiatric Association, 1987)

DSM IV: Diagnostic and Statistical Manual of Mental Disorders, 4th Edition (American Psychiatric Association, 1994)

DSM 5: Diagnostic and Statistical Manual of Mental Disorders, 5th Edition (American Psychiatric Association, 2013)

ADI-R: Autism Diagnostic Interview Revised (Lord et al., 1994)

ADOS: Autism Diagnostic Observation Schedule (Lord, Rutter, Goode, 1989)

CARS: Childhood Autism Rating Scale (Schopler, Reichler, DeVellis, Dal, 1980)

CARS-2: Childhood Autism Rating Scale, Second Edition (Schopler, Van Bourgondien, Wellman, 2010)

CASI-4R: Child and Adolescent Children Inventory, 4th Edition, Revised (Gadow & Sprafkin, 2005)

ASQ: Autism Spectrum Quotient (Baron-Cohen et al., 2001)

SRS: Social Responsiveness Scale (Constantino et al., 2003)

ABC: Autism Behaviour Checklist (Krug et al., 1978)

ILAE 1981: International League Against Epilepsy (Commission on Classification and Terminology, International League Against Epilepsy, 1981)

ILAE 1989: International League Against Epilepsy (Commission on Classification and Terminology, International League Against Epilepsy, 1989)

ILAE 2005–2009: International League Against Epilepsy, 2005–2009 (Berg et al., 2010)

NHIS 2013: National Health Interview Survey (National Center for Health Statistics. National Health Interview Survey, 2014)

GPRD: The General Practice Research Database (Kaye, Melero-Montes, Jick, 2001)

**Table 2:** Study characteristics for case control studies

Authors	Year	Country	ASD SS	M	F	AGE m,(r)	CGS	CGT	Sample Source	ASD + E SS	Epilepsy Prevalence	ASD Definition/ Instruments	Epilepsy Definition (criteria)	Study Quality	
Moon et al.	2020	Singapore	128	109	19	• ASD alone: 22.1, (16.7–27.5) • ASD + ID: 23.2, (15-8-30.6)	144	Adults with ID	Clinical Sample	10	7.8%	DSM 5	Clinical history	Moderate	
Zhang et al.	2019	China	192	146	46	8.20, (3–12 range)	74,059	Healthy Controls	Population Based	22	11.5%	DSM 5, SCQ	ICD 10, MRI	Moderate	
Weber et al.	2017	USA	589	486	103	9.5, (6–18)	653	Outpatient Psychiatry Referrals	Clinical Sample	43	7.3%	DSM IV, Developmental History, Clinical Interview with the caregiver, observations, ASD rating scales (CASI-4R), SCQ, ADOS)	Clinical Correlates and Somatic Conditions and Parents Questionnaire (Gadow et al. 2008)	Weak	
Su et al.	2016	Taiwan	7530	5930	1600	7.9, (0–18)	7530	Healthy Controls	Population Based	NR (incidence rate: 13.7)	NA	ICD9-R	ICD 9-R	Moderate	
Mouridsen et al.	2016	Denmark	118	85	33	4.9, (2–15)	336	Healthy Controls	Clinical Sample	29	24.6%	ICD 9	ICD 8 or 10	Moderate	
Ko et al.	2016	South Korea	66	64	2	• ASD only: 8.273, (NR) • ASD + Epilepsy: 10.227, (NR)	44	ASD without cooccurring epilepsy	Clinical Sample	22	33.3%	CARS, ADI-R	‘two or more non-febrile seizures that were not confined to pre-school period (up to 5 years) assessed via neurological assessment & ILAE 2005		
McCue et al.	2016	NR	610	477	73	8.48, (2–18)	160	Unaffected siblings	Clinical Sample	72	11.8%	DSM IV, ADOS, ADI-R	Retrospective Interview to retrieve Medical History	Moderate	
Bishop et al.	2016	USA	903	NR	NR	6.80, (NR)	282	Clinical Sample	Clinical Sample	85	9.4%	ADI-R, ADOS, DSM IV	Clinical history	Moderate	
Jokiranta et al.	2014	Finland	4393	NR	NR	NR	18,528	ASD without cooccurring epilepsy	Population Based	312	7.1%	ICD 9, ICD 10	ICD 9, ICD 10	Good	
Jain et al.	2014	USA	33,565	27,479	6086	NR, (0–20)	138, 876	Non-ASD on Health Care Plan	Population Based	2554	7.6%	ICD 9	Diagnostic codes in Medical Database	Good	
Valvo et al.	2013	Italy	206	174	32	7.1, (2.2-8)	134	ASD without cooccurring epilepsy	Clinical Sample	58	28.2%	DSM IV-T, ADOS-G	ILAE 1981, 1989 EEG recordings	Moderate	
Mouridsen et al.	2013	Denmark	4180	3431	749	NR	4016	ASD without cooccurring epilepsy	Population Based	164	3.9%	ICD 10	ICD 10	Moderate	
Peacock et al.	2012	USA	8398	6754	1644	NR, (2–17)	1,464,383	Non-ASD on Health Care Plan	Clinical Sample	1063	12.7%	ICD 9	Medical Records Review	Moderate	
Mouridsen et al.	2012	Denmark	118	85	33	42.7, (27.3–57.3)	336	Healthy Controls	Clinical Sample	29	24.6%	ICD 8	ICD 8	Moderate	

YR = Year

• ASD SS: ASD Sample size, M: Males, F: Females, m: Mean, r: Range, ASD SS: ASD Sample size, ASD + E SS = ASD and Epilepsy Sample size, CGS: Control group size, CGT: Control group type, NR: not reported, NA: Not applicable

ICD 8: International Classification of Disease, 8th Edition (World Health Organization, 1971)

ICD 9: International Classification of Disease, 9th Edition (World Health Organization, 1978)

ICD 10: International Classification of Disease, 10th Edition (World Health Organization, 1992)

DSM IV: Diagnostic and Statistical Manual of Mental Disorders, 4th Edition (American Psychiatric Association, 1994)

DSM 5: Diagnostic and Statistical Manual of Mental Disorders, 5th Edition (American Psychiatric Association, 2013)

ADI-R: Autism Diagnostic Interview Revised (Lord et al., 1994)

ADOS: Autism Diagnostic Observation Schedule (Lord, Rutter, Goode, 1989)

CARS: Childhood Autism Rating Scale (Schopler, Reichler, DeVellis, Dal, 1980)

SRS: Social Responsiveness Scale (Constantino et al., 2003)

SCQ: Social Communication Questionnaire (Berument et al., 1999)

CASI-4R: Child and Adolescent Children Inventory, 4th Edition, Revised (Gadow & Sprafkin, 2005)

### Study Quality

The quality of the studies reviewed was assessed by two independent reviewers (EZ and CR) on the basis of the National Institutes of Health (NIH) Quality Assessment Tool for Case-Control studies and Observational, Cohort and Cross-Sectional Studies (National Heart, Lung and Blood institute, https://www.nhlbi.nih.gov/health-topics/study-quality-assessment-tools accessed November 15th 2020). Results of the quality assessment including scoring criteria are described in Tables 1 and 2 and Supplements 3 and 4 for observational/ cross sectional/cohort studies and case control studies respectively.

**Table 3:** Factors linked to the co-occurrence of epilepsy in autism

Authors	YEAR	BD/PD	CL/ID	GEN	AGE	FC	I/A	REG	LANG	SS	MG	DW
Fombonne et al.	2020			NSIG								
Bishop et al.	2020		SIG	SIG	SIG							
Gilmore et al.	2020		SIG									
Moon	2020		SIG									
Zhang et al.	2019			SIG	SIG							
Thompson et al.	2019							NSIG				
Lamb et al.	2019			SIG				NSIG				
Zhang et al.	2018	NSIG			SIG		NSIG					
Weber et al.	2017		SIG	NSIG	NSIG	NSIG	NSIG					
Gadow et al.	2017							SIG				
Su et al.	2016	NSIG	SIG	NSIG	NSIG						NSIG	
Ko et al.	2016									SIG		
Wu et al.	2016			SIG								
McCue	2016				SIG							
Ayta et al.	2016							NSIG				
Christensen et al.	2016					SIG						
Mouridsen	2016						SIG					
Bishop et al.	2016											NSIG
Fortuna et al.	2016				SIG							
Shubrata et al.	2015								SIG	SIG		
Saltik et al.	2014											
Mulligan et al.	2014	SIG										
Jokirant et al.	2014		SIG									
Jain et al.	2014						SIG					
Doshi-Velez et al.	2014	NSIG										
Schuch et al.	2014										NSIG	
Mouridsen et al.	2013			NSIG								
Valvo et al.	2013	SIG				SIG		NSIG	NSIG			
Eriksson et al.	2013		SIG	SIG								
Saltik et al.	2012											SIG
Mouridsen et al.	2012						NSIG					
Ververi et al.	2012		NSIG									
Bolton et al.	2011		SIG	SIG					SIG			
Parmeggiani et al.	2010		SIG	NSIG	SIG		SIG				SIG	
Smith et al. A	2010	NSIG										
Smith et al. B	2010	NSIG										
Ming et al.	2008							NSIG				
Oslejskova et al.	2007							SIG	SIG			
Hara	2007		SIG	NSIG							NSIG	NSIG
Billstedt et al.	2007									SIG		
Danielsson et al.	2005		SIG									
Pavone et al.	2004		SIG									
Fombonne et al.	2004							NSIG				

* Current not lifetime

*Abbreviations: SIG = Significant, NSIG = Not Significant*, BD/PD = Behavioral Disorders/ Psychiatric disorders, CL = Cognitive level/ Intellectual Disability, GEN = Gender, FC = Family Characteristics, I/A = Injuries/ Accidents, REG = regression, LANG = Language, DW = Delayed walking, SS = Social skills, M/G = Medical Genetic

### Data synthesis

Data was synthesised based on Synthesis Without Meta-analysis (SWIM) (Campbell et al., 2020) guidelines (see SWIM checklist Supplement 5).

The factors chosen for inclusion in the synthesis were based on a thorough reading of all full-text articles included in the review by EZ and CR and a previous review and meta-analysis (Amiet et al., 2008). In the absence of consensus, inclusion was also discussed with the wider review team. All included studies had to have at least one outcome determined to be clinically relevant by the review team and the outcome had to have been considered using standard statistical analysis.

All included studies were tabulated and in the tables (Table 3, Supplement 7 or Supplement 8) it was indicated whether or not the factor had been considered in statistical analysis and if so whether it was found to be statistically significant/not significant in relation to the occurrence of epilepsy in autistic individuals. In all studies, factors associated with occurrence of epilepsy in autistic individuals were deemed ‘statistically significant’ at the p < 0.05 level.

Reporting of results in the text were prioritised first with respect to total number of studies where the factor had been considered and then with respect to the quality of these studies.

## Results

### Search results

**Fig. 1:**
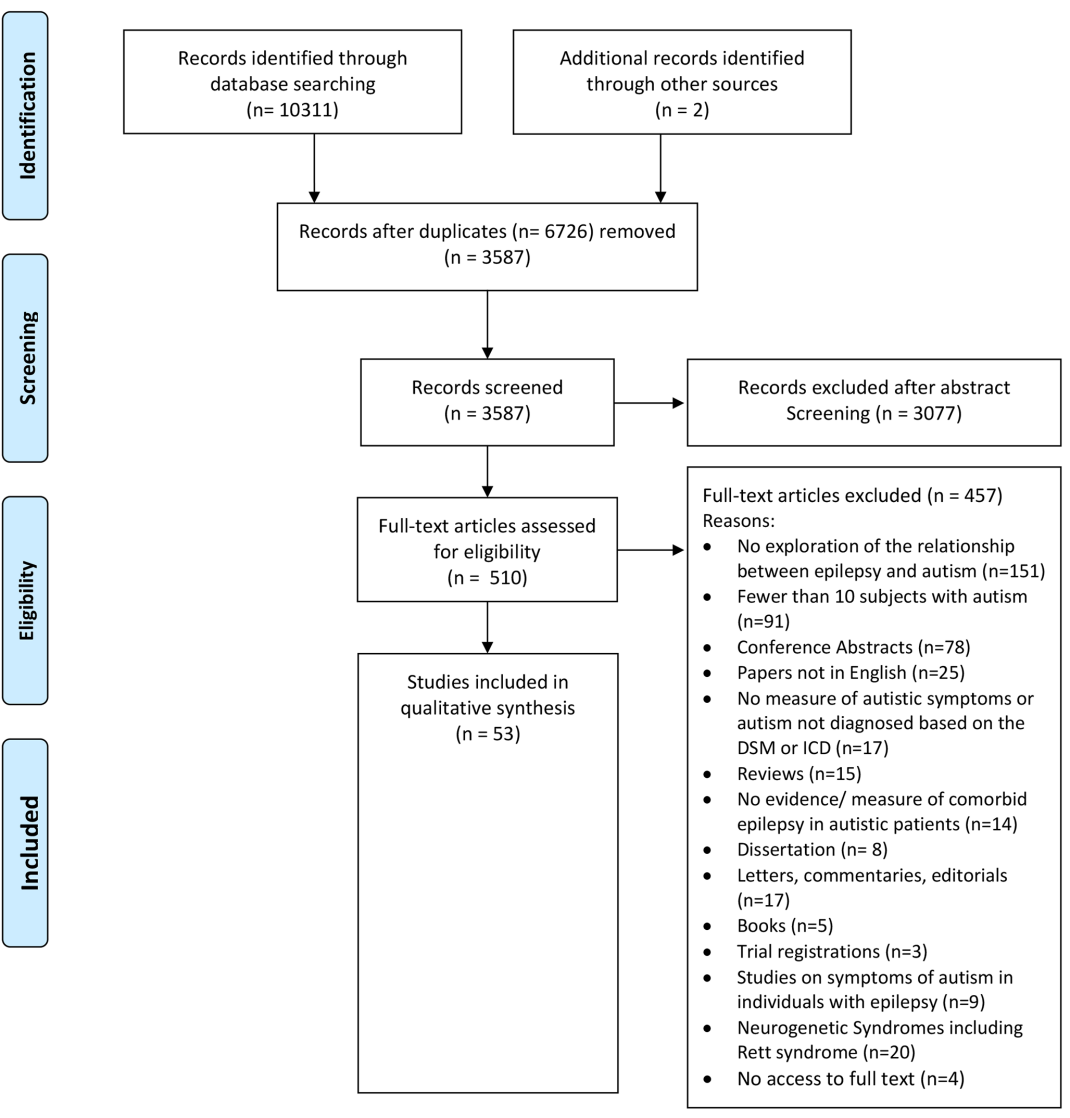
Prisma Diagram Search process for studies focusing on factors associated with epilepsy in autism

The initial search led to a total of 10,313 records, de-duplicated to 3,587, that were initially screened by two reviewers EZ and CR for relevance by ‘title’ and ‘abstract’. Of these, 3,077 were excluded while 510 were further assessed by full text against the exclusion and inclusion criteria (see Supplement 1). From this full text screening, 457 papers were excluded while the remaining 53 were deemed eligible for the present systematic review. A PRISMA diagram of the search and screening stages along with the reasons for exclusion are detailed in Fig. 1. The PRISMA checklist is in Supplement 6.

### Study characteristics

The 53 studies included in the present review included a total of 452,743 autistic individuals. Studies used the DSM-IV (n = 29) followed by the ICD-10 (n = 13), ICD-9 (n = 9), and the DSM-5 (n = 5) criteria to define the presence of autism, with some studies reporting multiple measures. Two studies (Billstedt et al., 2007; Pavone et al., 2004) used the DSM-III and one (Mouridsen et al., 2012) used the ICD-8.

All studies included more males than females. Three studies did not report on the gender distribution of the sample. From those that did, 67% were male (n = 90,934) while 33% were female (n = 45,423). Of the autistic individuals where the prevalence of epilepsy was reported (n = 257,892), 18,254 (7%) had co-occurring epilepsy. In studies which focussed on children with neurogenetic syndromes the prevalence of epilepsy in autistic individuals (n = 5,145) was 26%, highlighting that children with these syndromes are a particularly high-risk group for having co-occurring epilepsy.

Criteria used in the diagnosis of epilepsy included the ICD-8 (n = 2), the ICD-9 (n = 4), the ICD-10 (n = 9), International League Against Epilepsy (ILAE) 1981 criteria (n = 3), ILAE 1989 criteria (n = 5), ILAE (1993) (n = 1), ILAE 2005 criteria (n = 1) criteria, parent questionnaires (n = 6) and reviews of the patients’ medical data, clinical histories and EEG (n = 26), with some studies employing more than one method.

Studies were undertaken in 20 different countries across five continents: North America (19 studies), Europe (18 studies), Asia (12 studies), Oceania (2 studies) and Africa (1 study). The most common country was the USA (19 studies), followed by Denmark (4 studies) and the UK (4 studies). In one study the location was not specified.

Ten studies included adult participants only (19 years or older); 22 studies included child participants only (aged 18 years or younger); and 12 studies included both child and adult participants. In nine studies, participant mean age and range were not reported.

Study characteristics can be found in detail in Tables 1 and 2 referring to case-control studies and observational/cohort/cross-sectional studies respectively.

### Quality Assessment

Of the 39 observational, cohort and cross-sectional studies, seven were rated as ‘good’, 27 as ‘moderate’ and five as ‘weak’. The limited timeframe and lack of sample size justification were the most common reasons contributing to ‘moderate’ and ‘weak’ ratings in most studies (see supplement 3). Of the 14 case control studies, two were rated as ‘good’, 11 were rated as ‘moderate’ while the remaining one was deemed ‘weak’. All case control studies lacked sample size justification and blinding (see supplement 4). Detailed results of the quality assessments for observational, cohort and cross-sectional studies and case control studies can be found in supplements 3 and 4 respectively.

### Factors Associated with the co-occurrence of Epilepsy and Autism

Details on the factors associated with the co-occurrence of epilepsy and autism can be found in Table 3. Factors are included in the table if they were considered in three or more studies. All factors considered including those only considered in one or two studies are available in supplement 7.

The most frequently considered factor was cognitive level and the presence of intellectual disability (13 studies), followed by gender (12 studies), history of developmental regression (9 studies), the presence of behavioral/psychiatric disorder(s) (7 studies), chronological age (8 studies), injuries/accidents (5 studies) and language (6 studies). Medical/Genetic factors were considered in four studies. Social skills, delayed walking, and family characteristics were considered in three studies. These are described in more detail below. All other factors were considered in two or fewer studies.

### Level of Cognition or Presence of Intellectual Disability

Lowered cognitive level or the presence of intellectual disability (ID) was significantly associated with occurrence of epilepsy in 12 of 13 studies where it was considered. The one study where the presence of epilepsy in autistic people was not found to be significantly associated with ID/level of cognition was Ververi et al., (2012). In this study of 222 autistic children aged 1.5-9 years, 17 (8%) had epilepsy and 51 (23%) children had ID. In nine of the 11 studies which found a significant association between epilepsy and ID/Lower level of cognition, the relationship was between presence of epilepsy and ID typically defined as IQ < 70 or as ID recorded in medical registers. In one study which did not employ ID status, Bolton et al., (2011) found that autistic people with epilepsy had significantly lower nonverbal ability IQ as measured by Ravens Matrices (Raven & Court, 1986) than autistic people without epilepsy. Additionally, Pavone et al., (2004) reported that in autistic children and young adults (4–21 years), epilepsy was significantly more common in individuals with IQ < 55 compared with IQ > 55. Parmeggiani et al., (2010) reported that autistic adults and children were more likely to have severe/profound ID (IQ less than 35) than autistic children and adults without epilepsy. Two other studies also reported that epilepsy was significantly more common in individuals with a greater degree of intellectual disability. Danielsson et al., (2005) reported that the cognitive level was significantly lower in adults with epilepsy compared to those without. Among the autistic people with severe ID (IQ < 50), 48% had epilepsy compared with 20% with Mild ID (50–69) and 17% in autistic people without ID (IQ < 70) in this study. Jokiranta et al., (2014) noted that the risk for epilepsy in autistic people significantly increased among more severe cases of ID. Four of the thirteen studies which considered the role of ID/ lowered cognition were considered ‘good’ quality and in all of these studies a lower level of cognition or the presence of ID was associated with an increased occurrence of epilepsy in autistic individuals.

### Gender

Gender was a significantly associated with the occurrence of epilepsy in six of 12 studies. For all significant findings with the exception of one study, female gender was associated with an increased risk of the occurrence of epilepsy in autistic individuals. The one exception was Wu et al., (2016) who found an increased risk for epilepsy in males with autism. Study quality was deemed ‘good’ in two of the studies with significant findings (both showed an increased occurrence of epilepsy in female autistic individuals) and ‘moderate’ in the other studies with significant findings.

### Developmental Regression

The presence of developmental regression was significantly associated with an increased occurrence of epilepsy in autistic individuals in three of nine studies and not significantly associated in the other six studies. One of the studies which found an increased occurrence of epilepsy in autistic individuals had a ‘good’ rating whilst the other two had a ‘moderate’ rating.

### Behavioral/Psychiatric Disorders

The presence of additional behavioral or psychiatric disorders were significantly associated with having epilepsy for autistic individuals in two of the six studies where it was considered. With respect to the type of behavioral/psychiatric disorders considered, the studies tended to combine disorders or included results of broad-based rating scales so no clear patterns with respect to which type of difficulty/disorder might be associated with an increased occurrence of epilepsy in autism were evident. One of the studies which considered the presence of behavioral/psychiatric disorders was considered ‘good’ and it found a significantly increased occurrence of epilepsy in autistic individuals with additional behavioral/psychiatric disorders.

### Chronological age

Chronological age was significantly associated with the occurrence of epilepsy in autistic individuals in six of eight studies where it was considered. In five studies increasing age was associated with an increased occurrence of epilepsy in autistic indivdiuals. Two of these studies focused on child populations, two on adult populations and one on a mixed child/adult population. Zhang et al., (2018) found that autistic children aged between 13 and 17 years were more likely to have epilepsy compared with younger children (under 13). McCue et al., (2016) conducted analysis of data from a registry based retrospective cohort study of 731 autistic children (2–18 years) and increasing age was significantly associated with the presence of non-febrile (i.e. epileptic) seizures. Fortuna et al. (2016) collected cross-sectional data on 255 autistic adults aged 18 to 71. Compared to younger autistic adults (aged 18–29), autistic adults aged 40 years and older had an increased prevalence of epilepsy but not compared with those aged 30–39. Bishop et al., (2020) found that for autistic adults there was increasing prevalence with increasing age (until the oldest category (60 years+) in which death likely attenuated estimates. There was however, no association between age and epilepsy prevalence in autistic adults without ID. Parmeggiani et al., (2010) included both autistic adults and children (aged 2–37 years) and noted and a significant association between epilepsy and increasing age. In one study, younger age (3-5years as opposed to 7–9 and 10-12years) was more strongly associated with an increased occurrence of epilepsy (Zhang et al., 2019). None of the studies which considered age received a ‘good’ quality rating. Interestingly, Hara (2007) noted that the median age for epilepsy onset in a sample of 130 autistic adults (18–35 years) was 14 years whilst Parmeggiani et al., (2010) suggested that age of onset of seizures had two peaks (0–5 years and between 10 and 15 years). However, no statistical analyses were reported for these findings.

### Injuries/Accidents

The presence of injuries or accidents was significant in two of the five studies where it was considered and not significant in the other three studies. In the two studies where it was significant there was an association between the presence of accidents/injures and an increased risk for epilepsy in autistic individuals. One of the studies where an increased risk for epilepsy was found had a ‘good’ rating whilst the other four studies were ‘moderate’ or ‘weak’.

### Language Difficulties

Difficulties with language was associated with the occurrence of epilepsy in autistic individuals in three of the four studies where it was considered. Two of these three studies where a significant association with language difficulties and the occurrence of epilepsy in autistic individuals was found were considered ‘good’ quality and both noted a significant relationship between language difficulties and the co-occurrence of epilepsy and autism. Bolton et al., (2011) followed up 150 autistic individuals diagnosed in childhood at 21 + years. 33 (22%) had developed epilepsy. Language was measured via items from the ADI-R (Rutter et al., 2003) including ‘loss of language skills’and ‘overall level of language’, and the British Picture Vocabulary Scale (BPVS) (Dunn et al. 1982). Epilepsy was significantly more common in individuals with very limited overall level of language and those who had significantly lower verbal ability measured using the BPVS. Oslejeskova et al. (2007) reported on 205 autistic children of whom 64 (31%) had epileptic seizures. Categorization of speech impairment was performed in collaboration with a speech therapist. Epilepsy in autistic children was associated with delay in development of speech as well as with severe language impairment /no development of speech. Shubrata et al., (2015) found that the autistic with children with epilepsy had a greater level of impairment on the Speech and Language domain of the PDD Assessment scale (Grossman R. The PDD Assessment Scale/screening questionnaire 2000 (Available at: http://www.infantsandchildren.net/wp-content/uploads/2009/06/website-autism-formfinal2.pdf) than autistic children without epilepsy. However, Valvo et al. (2016) did not find a relationship between the presence of epilepsy and expressive language development assessed through clinical observation in autistic people (2-21years).

### Delayed Walking

Delayed walking was found to be significantly associated with the occurrence of epilepsy in autistic individuals in one of the three studies where it was considered. Bishop et al., (2016) measured delayed walking using information about age of walking from question 5 on the ADI-R (Rutter et al., 2003) which asks “At what age did [subject] walk without holding on?”. Delayed walking was not associated with presence of seizures in the autistic individuals (aged 4–12 years). Saltik et al.’s (2012) definition of delayed walking was based on data from medical records and parental interviews. Autistic people with epilepsy (34%) had a higher rate of delay in onset of independent walking (later than age 18 months) as compared to autistic people without epilepsy (14%). Hara (2007) did not find a relationship between ‘age of walking alone’ and occurrence of epilepsy in autistic adults. All three studies received a ‘moderate’ quality rating.

### Social Skills

A greater level of impaired social skills in autistic individuals was associated with an increased occurrence of epilepsy in all three studies where it was considered and one of these studies received a good quality rating. Ko et al., (2016) found that autistic children with epilepsy scored significantly higher on the total score of the Social Responsiveness Scale (SRS; Constanino et al. 2003) than autistic children without epilepsy. Similarly, Shubrata et al., (2015) found that the autistic children with epilepsy had a greater level of impairment on the Social interaction subscale of the PDD Assessment scale (Grossman R. The PDD Assessment Scale/screening questionnaire 2000 (Available at: http://www.infantsandchildren.net/wp-content/uploads/2009/06/website-autism-formfinal2.pdf). Billstedt et al., (2007) found that presence of early onset epilepsy was associated with more impairment on the social interaction items of the DISCO (Diagnostic Interview for Social and Communication disorders; Wing et al. 2002) interview in a follow-up study of autistic adults diagnosed with autism as children.

### Family characteristics

Family characteristics were considered in three studies and found to be significant in two studies one of which received a ‘good’ quality rating. In one of the studies with significant findings, an older sibling having autism, an older sibling having epilepsy or an older sibling having both autism and autism were significantly linked to the co-occurrence of epilepsy in autistic individuals (Christensen et al., 2016). In the other study family history of seizures was significantly associated with the co-occurrence of epilepsy (Valvo et al., 2013).

### Medical/Genetic factors and Epilepsy in autistic individuals with Neurogenetic Conditions

Medical/Genetic Factors were considered in four studies and were found to be significant in one study. Parmeggiani et al., (2010) found that autistic children and adults with epilepsy had a significantly greater occurrence in cerebral lesions than those without epilepsy. Hara (2007) did not find that birth weight was associated with the co-occurrence of autism and epilepsy, whilst Su et al., (2016) did not find the presence of meningitis and Schuch et al., (2014) the presence of β3 integrin gene variants significantly associated with the co-occurrence. Details of studies where epilepsy was reported in autistic individuals with neurogenetic conditions are in supplement 9. The only factor found to be significantly associated with the co-occurrence was the presence of intellectual disability or lower cognitive level.

### Factors found to be significantly associated with the occurrence of epilepsy in autistic individuals in ‘good’ quality studies

All factors associated with the occurrence of epilepsy in individuals with autism in ‘good’ quality studies are shown in supplement 8. Regarding factors considered in less than two ‘good’ quality studies, a significant association between occurrence with epilepsy in autistic individuals was found for severity of autism symptoms (Pavone et al., 2004), increased hospitalisations (Jain et al., 2014) and lowered adaptive behavior (Danielsson et al., 2005).

## Discussion

The results of this systematic review suggest that having an intellectual disability or cognitive impairment is the most well-studied and the most frequently associated factor with the occurrence of epilepsy in autistic individuals. A range of other outcomes including female gender, presence of psychiatric/behavioral disorders and older age have been found to be associated with the occurrence, but not in all studies where they have been considered. Additionally, some features including language difficulties and a greater degree of social impairment have been considered less frequently but have been found to be associated with the occurrence in most of the studies where they have been considered. However, these associations were almost universally considered in the absence of attention to the role of intellectual functioning. In general, study quality was frequently ‘weak’ or ‘moderate’ meaning that there is a need for more robust designs including longitudinal studies to better elucidate why certain autistic individuals are at higher risk for epilepsy.

The increased association between epilepsy and autism in individuals with intellectual disability was noted in a previous systematic review (Amiet et al., 2008). When epilepsy and autism coexist, they likely share common pathophysiological mechanisms (Tuchman, 2017), and it is also likely that the occurrence of the two conditions in individuals with intellectual disability involves shared mechanisms for all three conditions (Tuchman, 2017). The mechanisms underlying the increased risks for the co-occurrence of the three conditions are likely to include both environmental and genetic factors (Besag, 2018). We identified a much higher prevalence of epilepsy in autistic individuals who have neurogenetic syndromes associated with intellectual disability (Supplement 9) highlighting that children with genetic syndromes associated with both autism and intellectual disability are a particularly high-risk group for co-occurring epilepsy. This is likely to reflect a shared pathophysiology and impact on early brain development manifesting as epilepsy, autism and intellectual disability. Studies of these conditions have revealed that in some cases early treatments may reduce seizures and intellectual impairments (O’Callaghan et al. 2018; Kotulska et al., 2021) but as of yet there is little data regarding autistic symptoms.

Males are at higher risk for autism than females, but this higher risk is attenuated in individuals with epilepsy (Lukmanji et al., 2019). The results of the current study suggest that female gender may even be a risk factor for the occurrence of epilepsy in individuals with autism. An issue with regard to gender distribution in populations of autistic individuals is the increasing recognition that autistic females have historically been under recognized (Mandy et al., 2012) and this could lead to ascertainment bias in that autistic males are more likely to be diagnosed than females (Schuck et al., 2019). This under recognition of females may also impact the gender distribution with respect to the co-occurrence of both epilepsy and autism.

The co-occurrence of behavioral/psychiatric disorders in individuals with autism is common, affecting more than 70% of individuals with autism (Simonoff et al., 2008), and results of the current review suggest that this occurrence may also increase the risk for epilepsy. Studies have predominantly not focused on individual psychiatric/behavioral disorders, so it is not clear if it is a general risk or a more specific risk. Studies of individuals with ADHD suggest a similar or slightly increased occurrence for epilepsy compared to the non-ADHD population (Socanksi et al. 2013; Davis et al., 2010) but the prevalence is lower than that observed in autistic individuals. Reasons for the higher prevalence of epilepsy in autism compared with ADHD could include that the genetic risk for autism and epilepsy is shared with intellectual disability whereas the risk for ADHD and epilepsy is somewhat different. It will, therefore, be useful to consider the occurrence of epilepsy in individuals with both autism and ADHD as opposed to just autism alone whilst also considering the role of intellectual functioning. Future studies should also examine the association between epilepsy and autism with respect to disorders such as depression likely to emerge in older children and adults.

It has been claimed that ‘developmental regression’ occurs in approximately one in five autistic individuals and in half of these the ‘regression’ is from typical development (Thompson et al. 2019). Developmental regression was significantly associated with occurrence of epilepsy in three of nine studies in the current review. Differences between study samples but also definitions of regression may have led to this mixed finding. It is important that in future studies ‘regression’ is clearly defined to identify whether children with autism who lose or have lost skills have an increased occurrence of epilepsy.

In the current study increasing age was found to be associated with an increased risk for the occurrence of epilepsy in some but not all studies where it was considered. It has been suggested that there may be two peak periods for the development of epilepsy in autism, namely early childhood and adolescence (Hara et al. 2006; Parmegiani et al. 2010), although this second peak in adolescence was not noted in one longitudinal study (Danielsson et al., 2005). Differences between findings are likely to reflect study design but also definitions of both epilepsy and ‘remission’ from epilepsy. More longitudinal studies, which employ accepted definitions of epilepsy and remission, but which also track autism symptoms over time are needed to determine if age of onset of epilepsy in the autistic population is actually different from the onset of epilepsy in the non-autistic population.

Difficulties in language or verbal abilities was significantly associated with the occurrence of epilepsy in some autistic individuals. However, delays or difficulties in language have predominantly been considered using univariable statistical methods and intellectual ability has not been reported or not considered in statistical analysis. It is therefore, unclear if specific difficulties in language are independently associated with an increased occurrence of epilepsy in individuals with epilepsy or whether the co-occurrence is predominantly driven by broader intellectual difficulties. Similarly, it is not known if more difficulties in social skills or delayed walking in individuals with autism is associated with an increased risk for epilepsy independently of difficulties in intellectual functioning.

### Future research directions

Study quality was mixed with only one in five of included studies receiving a ‘good’ rating. This highlights the need for more robust study designs. The increase in the incidence and prevalence of registered diagnosed of autism likely due to rising diagnosis among adults, females and higher functioning individuals (Russell et al., 2021; Lundström et al., 2015) highlights the need to consider that cohorts in older studies may not be representative of current diagnostic practices. The use of longitudinal designs following autistic individuals into adulthood will be helpful. Additionally, following children at high risk for autism such as siblings, as well as following children with early onset seizures in the first two years of life will also be helpful in establishing what factors might play a role in the development of epilepsy and autism at different time periods. It is particularly important to use multivariable analysis methods to understand whether language difficulties, motor difficulties or ADHD symptoms are associated with the occurrence of epilepsy in autistic individuals independent of global intellectual impairment. More research is also needed from low- and middle-income countries given the higher incidence of epilepsy but also more challenging conditions with respect to screening and diagnosis in these settings. Additionally, there is a paucity of studies considering the economic impact of having both epilepsy and autism and studies of this nature are needed.

### Limitations

We excluded articles not in the English language and could not obtain full texts for a small number of articles which may have been relevant. We did not explore heterogeneity with respect to reported statistical methods, but this was considered in our quality analysis. Employing meta-analytic methods may have yielded more objective results with respect to the nature of relationships between factors associated with the occurrence of epilepsy in autistic individuals. We chose to exclude studies published prior to 2000 as it was felt studies undertaken before this may not reflect the broadening of the concept of autism evident since the publication of DSM-IV in 1994 (American Psychiatric Association, 1994). The chosen exclusion cut-off of prior to 2000 is arbitrary and may have influenced our findings. In relation to autism, we included only studies where individuals were diagnosed with respect to DSM/ICD criteria. However, our inclusion criteria for individuals diagnosed with epilepsy was not as stringent and this may also have impacted on findings. Although we provide basic details of studies that included predominantly participants with neurogenetic syndromes a thorough discussion of neurogenetic conditions associated with autism and epilepsy is beyond the scope of this systematic review.

## Conclusions

A wide range of factors were considered with respect to possible factors associated with the occurrence of epilepsy in autistic individuals. The presence of intellectual disability or cognitive impairment is the factor most consistently associated with this occurrence. A number of other factors are potentially important with respect to the occurrence, but study quality and lack of significant findings in all studies where these other factors have been considered means better quality research is needed to establish what factors independent of intellectual impairment are important in the co-occurrence.
